# Toward high-throughput engineering techniques for improving CAR intracellular signaling domains

**DOI:** 10.3389/fbioe.2023.1101122

**Published:** 2023-03-27

**Authors:** Savannah E. Butler, Colin J. Hartman, Yina H. Huang, Margaret E. Ackerman

**Affiliations:** ^1^ Department of Microbiology and Immunology, Geisel School of Medicine at Dartmouth, Hanover, NH, United States; ^2^ Thayer School of Engineering, Dartmouth College, Hanover, NH, United States; ^3^ Department of Pathology and Laboratory Medicine, Geisel School of Medicine at Dartmouth, Hanover, NH, United States

**Keywords:** chimeric antigen receptor, OX40, 41BB, directed evolution, mammalian display

## Abstract

Chimeric antigen receptors (CAR) are generated by linking extracellular antigen recognition domains with one or more intracellular signaling domains derived from the T-cell receptor complex or various co-stimulatory receptors. The choice and relative positioning of signaling domains help to determine chimeric antigen receptors T-cell activity and fate *in vivo*. While prior studies have focused on optimizing signaling power through combinatorial investigation of native intracellular signaling domains in modular fashion, few have investigated the prospect of sequence engineering within domains. Here, we sought to develop a novel *in situ* screening method that could permit deployment of directed evolution approaches to identify intracellular domain variants that drive selective induction of transcription factors. To accomplish this goal, we evaluated a screening approach based on the activation of a human NF-κB and NFAT reporter T-cell line for the isolation of mutations that directly impact T cell activation *in vitro*. As a proof-of-concept, a model library of chimeric antigen receptors signaling domain variants was constructed and used to demonstrate the ability to discern amongst chimeric antigen receptors containing different co-stimulatory domains. A rare, higher-signaling variant with frequency as low as 1 in 1000 could be identified in a high throughput setting. Collectively, this work highlights both prospects and limitations of novel mammalian display methods for chimeric antigen receptors signaling domain discovery and points to potential strategies for future chimeric antigen receptors development.

## Introduction

CAR T-cell therapy has shown remarkable success in treating hematological cancers (Han et al., 2021). However, this success has not extended to solid cancer treatment, where efficacy is thought to be compromised by off-tumor toxicity, poor trafficking to the tumor site, limited persistence and proliferation, and functional suppression within the tumor microenvironment (Rafiq et al., 2020; Marofi et al., 2021; Sterner and Sterner, 2021). These barriers point to the need for further improvements in CAR T-cell design in order to more fully realize the clinical potential of targeted T cell therapies against more challenging tumor types.

To this end, while optimization of extracellular domains has benefitted from a diversity of high-throughput strategies used widely to affinity mature candidate receptors for antigen recognition (Rydzek et al., 2019; Bloemberg et al., 2020; Wang et al., 2021), other factors appear to limit *in vivo* performance. Some of these factors are beginning to be addressed by engineering expression of additional immune modulators, such as programming secretion of cytokines, and employing ON or AND switches, etc., (Rafiq et al., 2020). However, improvements in CAR activity and persistence are still likely to benefit from continued optimization of their primary sequence and structure. To efficiently explore this design space, higher throughput approaches are needed. Presently, CAR T cell design strategies generally evaluate novel CARs on a candidate-by-candidate basis, in which constructs are designed and tested one at a time based on a hypothesis about *in situ* performance. Candidates are then prioritized based on performance in parallel assays of varying throughput. While this rational design approach has produced the successful CAR designs used in the clinic today, more efficient and less modular discovery and optimization approaches could enable faster CAR screening, allow evaluation of greater functional diversity and T-cell fitness, and potentially accelerate CAR therapeutic design for improved clinical success rates.

As has been done for small molecule and antibody drug discovery, high-throughput screening approaches could permit deployment of directed evolution strategies. While such approaches have successfully identified novel extracellular, antigen binding domains (Han et al., 2017; Hua et al., 2017; Sharma et al., 2020; Butler et al., 2022), analogous strategies are only beginning to be developed for evaluating intracellular signaling domains in the context of a complete CAR construct, in T cells stimulated by target antigen, and which allow selection of functional phenotypes. Among CAR domains, signaling domains pose unique challenges to the binding affinity-based approaches common in high-throughput directed evolution. Unlike extracellular domains, intracellular signaling domains rely on dynamic posttranslational modifications and spatial positioning of signaling mediators unique to T-cell biology.

CARs contain the TCR-associated CD3ζ intracellular domain and are thus expected to trigger similar signaling cascades as natural TCR signaling. Indeed, both CAR and TCR activate canonical downstream pathways including Lck and Zap-70 kinases, LAT, and PLCγ1 (Salter et al., 2018). However, recent studies have revealed that CARs and TCRs exhibit distinct activation thresholds (Harris et al., 2018; Watanabe et al., 2018), downstream phosphorylation kinetics (Karlsson et al., 2015; Rohrs et al., 2018; Salter et al., 2018; Ramello et al., 2019), and recruitment of negative regulators (Hamieh et al., 2019; Sun et al., 2020). Altered signaling may be attributable to the tandem linkage of several signaling domains in CARs. This may remove normal barriers imposed by spatial separation of endogenous receptors and co-receptors but may also disrupt evolutionarily optimized spatial pairing of signaling partners. For example, fusing the CD28 co-receptor sequence to the TCR CD3ζ sequence increases the rate of CAR phosphorylation at its CD3ζ motifs (Rohrs et al., 2018). Yet, swapping domain order alters the magnitude of cytokine production and tonic signaling (Guedan et al., 2018). Additionally, domains typically expressed in other immune cells, such as the NK-cell signaling adapter DAP10, exhibit unique properties when incorporated into CAR T cell designs (Zhao et al., 2019). The cumulative result of this complexity is that the specificity and contributions of individual costimulatory domains is experimentally difficult to determine (Jutz et al., 2016).

Importantly, while modular design approaches will continue to improve our understanding and lead to more successful future CAR constructs, current strategies rely predominantly on wildtype domains, leaving the vast mutational landscape of each domain unexplored. Candidate signaling domains poised for high throughput optimization include existing T cell signaling domains used in preclinical and clinical trials, CD28, 4-1BB, OX40, and CD3ζ (Gilham et al., 2002; Carpenito et al., 2009; Savoldo et al., 2011). Among these, OX40 and 4-1BB are co-stimulatory molecules in the TNF-receptor (TNFR) family. When incorporated into CARs, OX40 promotes T cell adhesion to endothelial cells as well as proliferation and differentiation (Hombach et al., 2012), while 4-1BB prolongs survival of activated T cells and enhances IL-2 production (Long et al., 2015). Both co-stimulatory domains lack intrinsic enzymatic activity and rely on binding to TNF Receptor Associated Factor (TRAF) adapter proteins in order to transduce downstream signals (Xie, 2013). Upon receptor ligation, TRAF proteins are recruited as homo- and/or heterotrimers to the 4-1BB or OX40 intracellular domain, initiating the formation of the signalosome (Zapata et al., 2018). There are six known TRAF proteins (TRAF1-TRAF6) that are regulated by expression and subcellular localization in the cell. Thus, the composition of TRAF homo- and heterodimers is dependent on cell type and activation state. While OX40 and 4-1BB bind to multiple TRAF proteins, TRAF2 binding has been linked to canonical NF-κB signaling and cell survival (Etemadi et al., 2015). OX40 and 4-1BB interact with low affinity to TRAF2 (50 µM and 1 mM, respectively) (Ye and Wu, 2000), and while mutations that disrupt TRAF2 binding have been explored (Arch and Thompson, 1998), mutations that improve this interaction, and their effects on CAR phenotypes have yet to be described.

While affinity maturation of receptor interactions with downstream signaling partners may enhance signaling, it is also possible that affinity may be a poor proxy for activity, and that other attributes are more critical to improving CAR T cell function. Downstream of CAR signaling are the transcription factors NF-κB and NFAT, which regulate shared and distinct T cell effector functions. Moreover, NF-κB induction has been shown reflect the magnitude of T cell signaling (Berger et al., 2004; Kingeter et al., 2010; Thaker et al., 2015), while NFAT induction correlates positively with T cell stimulation (Clackson et al., 1998; Brogdon et al., 2002; Maguire et al., 2013). NF-κB and NFAT have both been used in reporter systems for the evaluation of TCRs and CARs (Jutz et al., 2016; Rosskopf et al., 2018; Rydzek et al., 2019). These studies suggest that an NF-κB and NFAT activation screen could be leveraged in a standardized directed evolution CAR discovery platform to identify novel signaling domains that induce physiologically relevant T cell effector responses. Here, we present a mammalian display approach capable of retrieving rare, functionally improved variants from among large libraries, which we anticipate will permit exploration of new terrain in the fitness landscape of CAR intracellular signaling domains.

## Materials and methods

### Protein production and purification

Soluble monomeric B7H6-His (accession number: NP_001189368.1) was expressed and purified as previously described (Butler et al., 2022). Briefly, the extracellular sequence of B7H6 with a 6xHis tag was cloned into the pCMV expression plasmid for transient transfection and expression in expi293 HEK cells (ThermoFisher). Soluble B7H6-His was purified from culture supernatant *via* nickel-charged immobilized metal affinity chromatography (ThermoFisher) according to the manufacturer’s instructions. Purified protein was filtered and stored in 1x PBS.

### Construction of J76 TPR cells

J76-TPR cells were a gift from Peter Steinberger at the Institute of Immunology in Vienna, Austria. Briefly, the TCR αβ negative Jurkat 76 cell line was transduced with NFAT-eGFP and NF-κB-CFP reporter constructs, and a single clone with low or absent expression of the reporter genes at steady state and high reporter gene expression upon stimulation with PMA/Ionomycin was used to generate the J76-TPR cell line, as previously described (Jutz et al., 2016; Rosskopf et al., 2018). J76-TPR cells were confirmed for eGFP and CFP expression by flow cytometry following stimulation with 5 ng/mL PMA and 400 ng/mL Ionomycin.

### Design and construction of CAR constructs

The CAR constructs were cloned into the pFB-Neo retroviral backbone and contained a single chain variable fragment (scFv) version of TZ47 (Hua et al., 2017), an antibody targeting the tumor antigen B7H6, the human CD28 transmembrane domain, either the human 4-1BB or Dap10 domain, the human CD3ζ cytoplasmic domain, a T2A sequence, and a truncated form of human CD19 (accession number: NM_001178098.2) used to monitor expression. Alanine substitution was performed using the QuikChange II site-directed mutagenesis kit (Agilent) following the manufacturer’s protocol. pFB-Neo retroviral control constructs were constructed with truncated human CD19 only in the backbone. Constructs were verified by Sanger sequencing.

### Retrovirus production and transduction of CAR-J76-TPR cells

HEK-293 T cells (2.5 × 10^6^) plated 18 h previously were transfected with pFB-Neo plasmid using Xfect Transfection Reagent (Takara Bio) following the manufacturer’s protocol. Media was replaced at 12 h post-transfection and supernatant was harvested at 48 h. To make stable cell populations which produce amphotropic virus for J76-TPR transduction, viral supernatant from HEK-293 T cells was passed through a 0.45 µm filter and then used to infect PG13 packaging cells in media containing 8 μg/mL of Polybrene. Amphotropic virus was collected, filtered, and used to generate CAR J76-TPR cells. J76-TPR cells were plated in a 24-well tissue treated plate (CytoOne) at a concentration of 1 × 10^6^ T cells per well in 1 mL of RPMI 1640 containing 10% FBS (ThermoFisher) and 1 mL of filtered viral supernatant was added to each well containing a total of 8 μg/mL Polybrene. Plates were spinoculated by centrifugation at 1,500 g for 60 min at 30°C and cultured 24 h before media replacing with fresh RPMI media. 2 days post transduction, G418 was added (1 mg/mL) and antibiotic selection continued for 5 days. On day 7, cells were analyzed for CD19 expression *via* flow cytometry, demonstrating expression in the majority but not across all cells in culture.

### CAR T cell stimulation

High-binding ELISA plates were coated with a serial dilution of soluble human B7H6-His starting at 50 ng/well in 50 µL of PBS overnight at 4°C. Following antigen incubation overnight, wells were washed twice with 1x PBS and blocked with 1% BSA PBS for 2 h at 37°C. After a final PBS wash, 5 × 10^5^ CAR J76-TPR cells were added to each well and cultured for 24 h at 37°C.

### Flow cytometry and enrichment analysis

4-1BB and 4-1BB K/O CAR J76-TPR cells were stimulated *via* plate-bound B7H6 as described above. Following stimulation, a fraction of each cell population was incubated with CellTracker™ Deep Red Dye (Invitrogen) following the manufacturer’s recommended conditions at a concentration of 1 µM. Labeled cells were then spiked into unlabeled 4-1BB K/O cells at a ratio of 1–10, 1 to 100, or 1 to 1000. Cells were stained with anti-hCD19 (Biolegend) for 1 h at RT in staining buffer (PBS with 2% FBS). Cells were then washed twice with staining buffer and were spun at 500 g for 5 min at RT. Samples were analyzed on a Miltenyi MACSQuant Analyzer 10 or an Agilent NovoCyte Advanteon. Enrichment ratio calculations were made with a minimum of 100 cells per cell type and based on a sort gate drawn to capture cells with both elevated NFAT and NF-κB activation signal. Following stimulation of 4-1BB, DAP10, and first-generation CAR J76-TPR cells with 50 μg/mL of plate-bound B7H6 as described above, activation of NF-κB and NFAT activity was measured *via* flow cytometry. Theoretical enrichments were calculated from unmixed cell populations by using the percentage of cells present in a sort gate based on elevated NFAT and NF-κB activation.

## Results

### Constructs to evaluate CAR-dependent NF-κB and NFAT activation in Jurkat T cells

**FIGURE 1:**
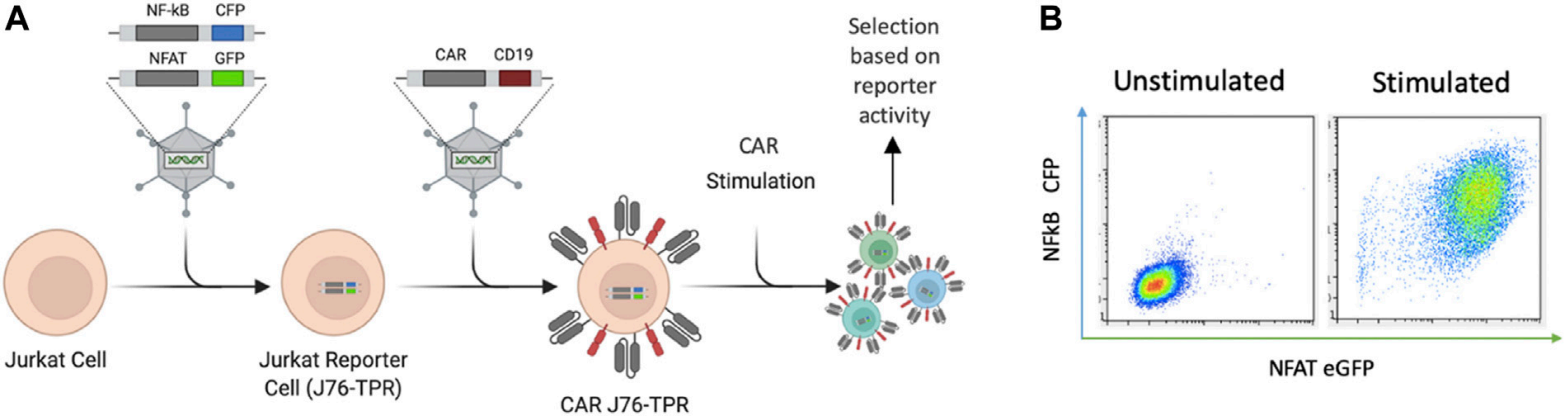
CAR-induced NF-κB and NFAT reporter construct activation in Jurkat cells. **(A)**. Schematic representation of reporter cells with NF-κB- and NFAT-inducible CFP and eGFP expression developed by Rosskopf et al., combined with retroviral CAR introduction to produce CAR J76-TPR cells. CAR constructs contain the positive selection marker hCD19. **(B)**. Representative flow biplots depicting fluorescence intensity of CFP and eGFP with (right) and without (left) stimulation of reporter cells with PMA and ionomycin (P/I). Panel A created in Biorender.

We sought to develop an *in situ* screening platform for the selection of signaling domain variants based on activation of downstream transcription factors associated with functional phenotypes. Screening variants in a T-cell line provides physiologically relevant expression and localization of signaling molecules critical for T cell signaling. Jurkat T cells were chosen for proof-of-concept experiments due to their ease of use, homogeneity, and availability of reporter cell lines. Importantly, the NF-κB and NFAT signaling machinery is highly conserved in the Jurkat cell line (Li and Verma, 2002). Others have previously shown that NF-κB and NFAT reporter genes allow for quantitative readouts of T cell activation by flow cytometry and demonstrated that this metric was able to distinguish between the quality of scFvs in a CAR library (Rydzek et al., 2019). We reasoned that this approach could also be used for differentiation among novel CAR intracellular signaling domains. The Jurkat reporter cell line (J76-TPR) is a TCR null variant of Jurkat E6.1 that expresses fluorescent reporter constructs containing response elements for NF-κB and NFAT (Figure 1A) (Rosskopf et al., 2018). Viral transduction of a library of CAR constructs in J76-TPR could be used as a mammalian display platform to identify CAR variants with desirable transcription factor activation profiles compared to maximal T cell activation following phorbol 12-myristate 13-acetate (PMA) and Ionomycin stimulation (Figure 1B).

### NF-κB and NFAT activation varies among different signaling domains

**FIGURE 2:**
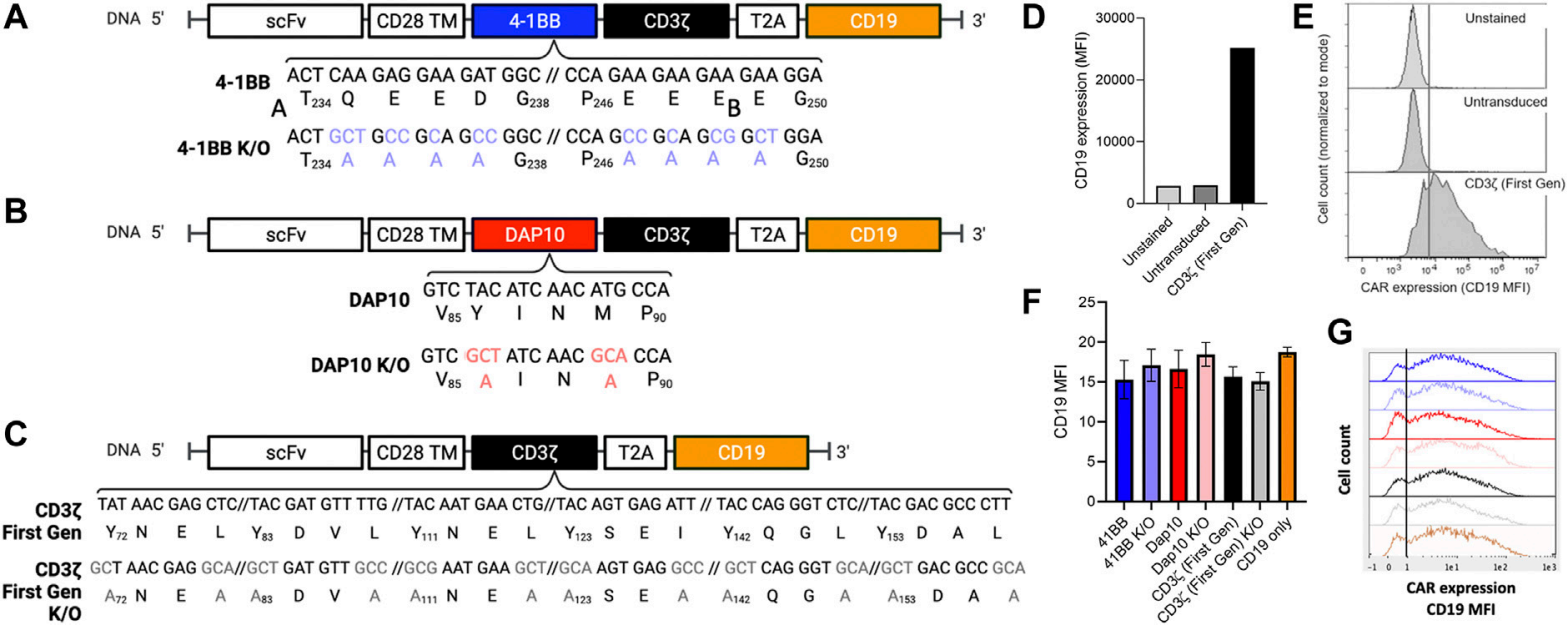
Positions, identities, and expression of 4-1BB, DAP10, and CD3ζ sequence variants. **(A)**. 4-1BB alanine substitutions of TRAF-binding residues. **(B)**. Dap10 alanine substitutions of YXXM residues, preventing phosphorylation and binding to PI3K and Grb2. **(C)**. CD3ζ alanine substitution of the ITAM sites, preventing phosphorylation and binding to Zap-70. **(D–G)**. Expression levels. **(D)**. Median Fluorescent Intensity (MFI) of the positive transduction marker, CD19, of unstained, untransduced and CD3ζ CAR-transduced cells serving as negative and positive controls, respectively. **(E)**. Representative CD19 fluorescent histograms for these positive and negative controls. **(F)**. Median Fluorescent Intensity (MFI) of the positive selection marker, CD19, of each CAR construct population following transduction. Error bars represent standard deviation of three technical replicates from one of three biological replicates. **(G)**. Representative CD19 fluorescence histograms of reporter cells transduced with these CAR constructs.

To determine if distinct signaling domains exhibit measurable differences in NF-κB and NFAT activation, second-generation CAR signaling variants were developed with 4-1BB or DAP10 co-stimulatory domains to evaluate distinct signaling modalities. DAP10 signaling is based on enzymatic phosphorylation while signaling through 4-1BB and other TNFR family members (4-1BB, OX40, etc.) are based on non-enzymatic TRAF binding. Additionally, CAR constructs containing loss-of-function mutations in 4-1BB and DAP10 were included to further investigate the ability of this screening platform to distinguish between mutations disrupting co-stimulatory but not primary signaling through the CD3ζ domain. We generated a 4-1BB mutant domain based on previously reported alanine substitution mutations for all amino acids in the TRAF binding site [(P/S/T/A)X(Q/E)E] to disrupt binding to downstream TRAF proteins (Ye et al., 1999) (Figure 2A). A DAP10 mutant domain was generated based on prior mutagenesis studies in which a M to A substitution in the YXXM motif disrupts binding to the p85 subunit of PI3K (Songyang et al., 1993) and a Y to A substitution in the YXXM/YXNX motif disrupts binding to both p85 and the Grb2 adapter protein (Upshaw et al., 2006) (Figure 2B). Because each second-generation CAR also contains a CD3ζ chain in addition to 41BB or DAP10, we also evaluated the degree to which CD3ζ alone contributes to NF-κB and NFAT activation by generating a first-generation CAR lacking a co-stimulatory domain. Lastly, a first generation CAR construct with Y to A and I/L to A substitutions in the CD3ζ ITAM motifs [YXX(I/L)] was generated to serve as a negative control (Figure 2C). Each CAR J76-TPR design included a truncated human CD19 sequence, following a T2A “self-cleaving” peptide sequence (Carey et al., 2009), which served as a marker of viral transduction efficiency (Wu et al., 2015) (Figure 2D). Although quite variable from cell to cell, CD19 expression was comparable (*p* > 0.05 by ANOVA with Dunn’s correction) among CAR construct populations (Figures 2E–G), thereby allowing us to directly compare reporter activity across CAR construct populations in the context of biological variability associated with site of integration, expression level, and other differences that impact stimulation and signaling, as would be anticipated in the context of library screening.

**FIGURE 3:**
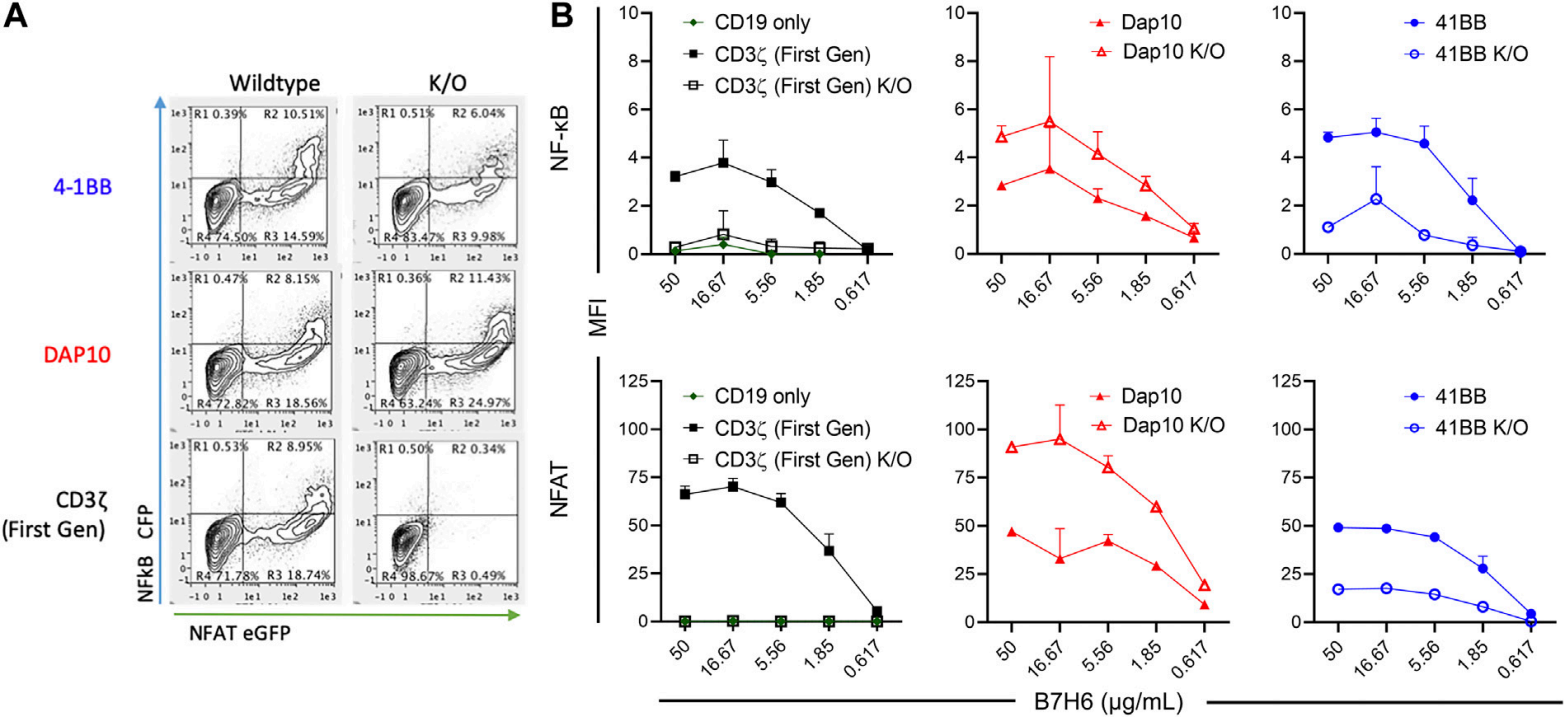
CAR-mediated activation of NF-κB and NFAT in signaling domain variants. **(A)**. Representative flow biplots of B7H6-stimulated (50 μg/mL) wildtype and K/O CARs showing activation of NF-κB and NFAT (left panel). **(B)**. MFI signal of NF-κB (top) and NFAT (bottom) in each CAR construct population across a titration of plated B7H6. Error bars represent standard deviation of three technical replicates from one of three biological replicates.

Each CAR-J76-TPR construct population was stimulated through the CAR by incubation with plate-bound tumor antigen B7H6 and evaluated for NF-κB and NFAT reporter activity by flow cytometry. J76-TPR cells expressing first-generation CAR induced NF-κB and NFAT reporter activity in an antigen dose-dependent manner (Figure 3). Neither the CD19 vector control nor first generation ITAM KO CAR-induced expression of either reporter. Among CAR constructs, the 4-1BB CAR induced the highest NF-κB reporter activity compared with first-generation or DAP10 CARs. However, disruption of the 4-1BB TRAF binding motif reduced NF-κB reporter activity compared to wildtype 4-1BB or first-generation CAR. The first-generation CAR unexpectedly exhibited higher dose-dependent NFAT signaling than either wild-type 4-1BB or DAP10 CARs (Figure 3). But disruption of the DAP10 YXXM motif resulted in higher DAP10 CAR induced NFAT reporter activity. Although overall NFAT activity was increased in DAP10 KO CAR designs, DAP10 WT had a higher subset of high functioning clones at every B7H6 concentration tested. These data indicate that mutations relevant to signaling domain function conferred distinct differences in NF-κB and NFAT signaling by 4-1BB and DAP10 second-generation and CD3ζ first-generation CARs. Moreover, they suggest that these screening parameters contain sufficient resolution to distinguish function-altering mutations from the wildtype sequences.

### Proof-of-concept enrichment of rare clones

**FIGURE 4:**
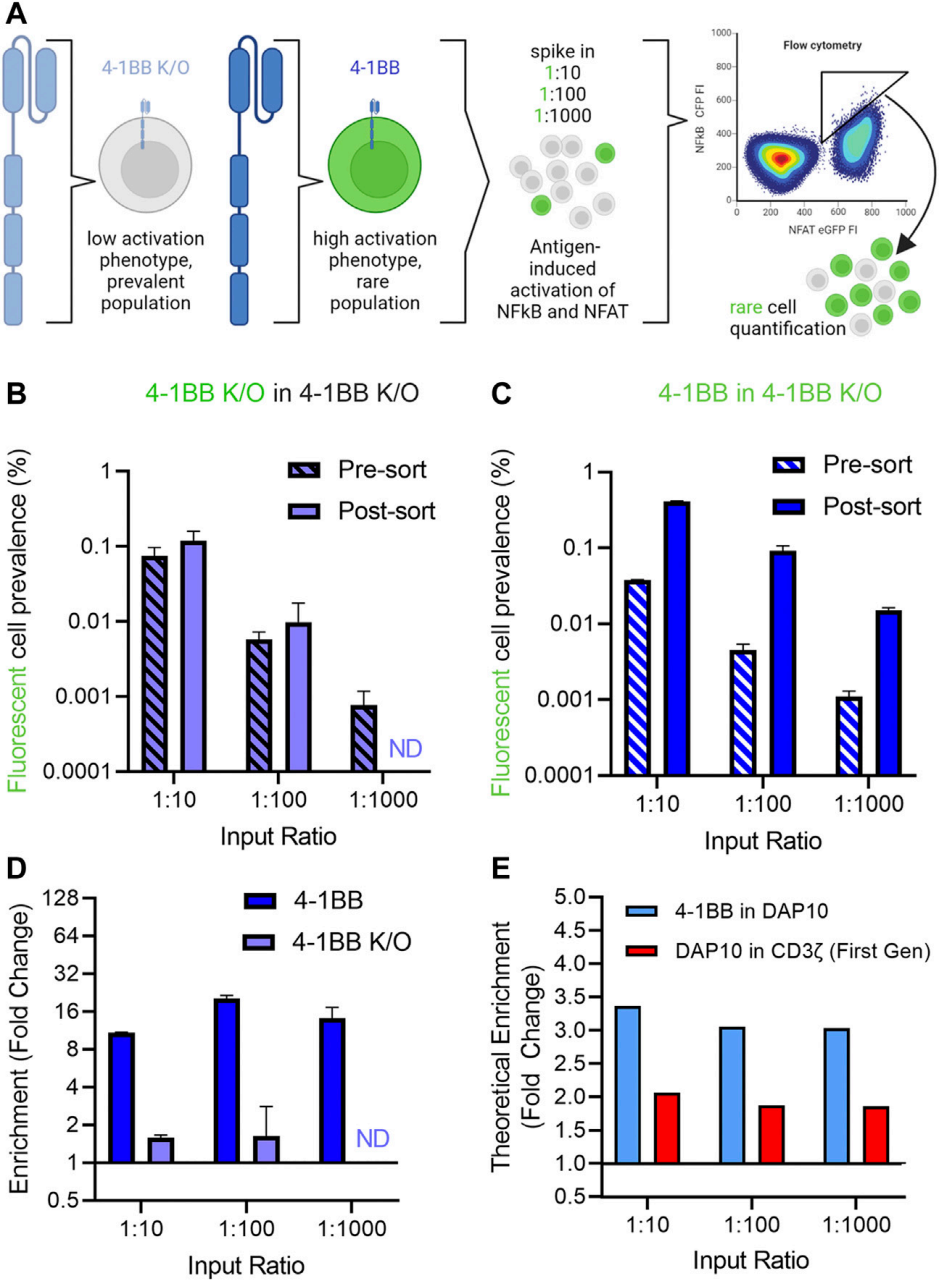
Enrichment of 4-1BB CAR reporter cells in a spike-in proof-of-concept mock sort. **(A)**. Schematic of needle in a haystack experiment to demonstrate the ability to retrieve rare variants that exhibit improved signaling profiles from among a larger population with reduced activity. **(B)**. Quantification of control fluorescent 4-1BB K/O cells spiked into an excess of non-fluorescent 4-1BB K/O cells before and after a mock selection based on NFAT and NFkB activation. **(C)**. Quantification of fluorescent 4-1BB cells spiked into an excess of non-fluorescent 4-1BB K/O cells before and after a mock selection for NFAT and NFkB activation for the 4-1BB spiked into 4-1BB K/O condition (right). Prevalence was calculated as the fraction of total CAR T cells as determined by CD19 expression. Pre-and-post sort labels refer to the mock sort on ungated and gated cells. **(D)**. Enrichment of cells was calculated by fold change in non-gated vs. gated populations. **(E)**. Theoretical enrichment was calculated using observed population distributions based on NFAT and NFkB activation of unmixed cell populations. Error bars represent standard deviation of three technical replicates from one of three biological replicates. Panel A created in Biorender.

Having established a screening platform capable of distinguishing signaling phenotypes from a panel of signaling domains, we next conducted a series of “needle in a haystack” experiments to investigate our ability to retrieve rare variants that exhibit improved signaling profiles from among a larger population with reduced activity (Figure 4A). 4-1BB CAR-J76-TPR cells were mixed with 4-1BB K/O CAR-J76-TPR cells at a ratio of 1:10, 1:100, and 1:1000 to enable evaluation of the effect of rarity on enrichment based on signaling phenotype. Prior to mixing, the rare cell population was stained with a fluorescent cell tracker dye to distinguish them from 4-1BB K/O cells in a mixed population. This strategy allowed us to determine the enrichment rate of rare cells in a simulated screen. As a negative control, a proportional fraction of fluorescently labeled 4-1BB K/O cells were spiked into non-labeled 4-1BB K/O cells.

While the fluorescently labeled control 4-1BB K/O were not enriched from among a larger number of unlabeled 4-1BB K/O cells (Figure 4B), 4-1BB cells were enriched from a population of 4-1BB K/O cells in all three ratio conditions when cells were sorted based on high NF-κB reporter activity (Figure 4C). Enrichment, calculated as the fold change in fluorescently labeled spiked in cells in the sort gate as compared to total cells, ranged from 10- to 20-fold across conditions (Figure 4D). The maximum enrichment was observed in the 1:100 condition, in which initial prevalence increased from 1 in 200 to 1 in 11 (20-fold enrichment). In each case, the level of enrichment observed experimentally was close to value expected given the phenotypic profiles of these populations in isolation. Thus, to better define the range of activity differences that could be differentiated, we calculated theoretical enrichment rates expected to result from a hypothetical sort gate applied to different mixed populations. Using this method, theoretical enrichment ratios of 2-4-fold were observed for 4-1BB spiked into DAP10 and DAP10 spiked into first generation CAR (Figure 4E), both of which exhibited more modest differences in activation phenotypes. This data demonstrates that despite cell-to-cell heterogeneity in CAR expression levels and stimulation, this selection platform can capture rare, higher signaling clones.

## Discussion

We explored a novel platform to investigate the functional landscape of intracellular signaling domains that would allow for the discovery of mutations that improve CAR dependent transcriptional activity and function. CAR signaling domain variants have recently been explored using exciting alternative strategies. Castellanos-Rueda et al. (2022) employed CRISPr-mediated insertion of domain shuffled CAR designs in primary T cells and single cell sequencing to screen CARs to identify novel intracellular domain configurations with distinct and desirable T cell transcriptional profiles. Analogously to this work, Gordon et al. (2022) used lentiviral transduction of CARPOOL, a CAR library of shuffled intact signaling domains, to identify desirable transcriptional and functional variants.

Here, to accomplish this objective, we evaluated an *in situ* screening strategy based on NF-κB and NFAT activation. While a number of excellent high-throughput methods exist for the engineering of high affinity protein-protein interactions to optimize CAR recognition of antigen, we sought a means to discover CAR variants that increase function but are more agnostic to means by which those increases are achieved. For example, design criteria for CAR intracellular domains are likely to relate less to affinity for signaling partners and more to factors that are challenging to capture in standard protein engineering systems, such as spatial orientation, signaling protein abundance and stoichiometry, and post-translational modification that impact the magnitude and kinetics of signaling. Differential kinetics of activation have been shown to contribute to different signaling fates (Salter et al., 2018). Thus, while it is clear that fine-tuning of intracellular domains through a rational design approach can lead to more desirable signaling outcomes (Feucht et al., 2019), and variations in these domains have improved next-generation CAR designs, robust coverage of design space has been challenging.

While no single *in vitro* activity is known to be perfectly predictive of CAR T clinical efficacy, several T cell characteristics have been tied to improved clinical outcome, including adhesion, migration, polyfunctionality, persistence, and proliferation (Rafiq et al., 2020; Marofi et al., 2021). Additionally, the transcription factors NF-κB and NFAT have both been shown to be reliable surrogates of T cell persistence and survival [(Maguire et al., 2013; Thaker et al., 2015)], (Brogdon et al., 2002; Song et al., 2008; Kingeter et al., 2010; Marangoni et al., 2013). Indeed, an NF-κB and NFAT reporter Jurkat cell line has demonstrated precedent as a successful screening tool for the selection of CARs with novel scFvs and hinge construction (Rydzek et al., 2019). However, to our knowledge this approach has yet to be adapted to intracellular CAR domains and the resolution of NF-κB and NFAT activation to detect meaningful differences driven by intracellular signaling domains is unknown. Here, we determined that this screening parameter possesses the capability to discern between 4-1BB, DAP10, CD3ζ CARs, and their respective functional knockouts.

Our data demonstrated that CD3ζ first generation CARs display a relatively high level of NFAT activation, perhaps an unexpected finding considering these CAR designs were retired due to their inability to promote complete T cell activation (Brocker and Karjalainen, 1995). The platform also allowed us to make the surprising observation that insertion of a mutated DAP10 motif further enhanced NF-κB and NFAT signaling compared to the first-generation CAR or native DAP10 CAR. This supports the importance of considering spatial orientation and membrane proximity of signaling components in CAR design. We also found that the 4-1BB CAR activates NF-κB and NFAT reporter activity to different degrees—enhancing NF-κB while reducing NFAT activity in comparison to the first-generation CAR. This observation demonstrates the platform’s capacity to identify CAR variants that uncouple downstream TCR signaling pathways. The ability of a limited screen to identify sequence changes that alter function in unexpected ways demonstrates the potential value of directed evolution over rational design for the engineering of these domains. While the optimal signaling and phenotypic profile of CAR T cells has yet to be fully defined, and likely varies across targets and tumor types, an ability to drive phenotypic divergence *in vitro* could contribute to functional differentiation of CAR designs that could then be screened *in vivo*.

Having established that NF-κB and NFAT signaling can be used to discern functional differences due to signal domain modifications, we tested the ability to retrieve rare, desirable CAR constructs from a larger population of undesirable CAR constructs. We found that the higher signaling cells were consistently enriched at three different dilution ratios, providing proof-of-concept that NF-κB and NFAT activation is capable of identifying high-signaling CAR designs even in the context of significant biological variability from cell to cell.

However, numerous technical hurdles remain for the establishment of a working display system for screening new CAR signaling variants using this approach. These include design and generation of a viral library of CAR signal variants of sufficient phenotypic variation to be reliably differentiated, optimization of antigen stimulation conditions to most thoroughly and uniformly stimulate a large number of cells, and recovery of selected cells and their CAR sequences either through regrowth or immediate lysis and capture of their genotypes, which can be complicated in the context of highly homologous sequences, necessitating either single cell approaches or barcoding of the library. While a number of non-viral CAR construct delivery alternatives could be pursued, optimization of each step is likely to contribute to setting the limits on feasible library sizes. For example, reliable readout of function may be complicated by multiple sequence integrations into one cell (Rydzek et al., 2019) and will need to be assessed in future studies. While viral transduction at a low multiplicity of infection may reduce multiple CAR sequence integration, we expect that such a step would also reduce the number of designs that could be screened for a given volume and number of cultured cells, or would motivate addition of a step in which CAR-expressing cells were first enriched prior to stimulation. For this reason, a mutagenesis strategy targeting known or suspected positions or domains of importance may be more appropriate than random mutagenesis of entire CAR constructs. We have also not experimentally confirmed how finely functional variants can be resolved but expect that biological noise between cells is likely to pose a fundamental limit given that even Jurkat T cells can exhibit heterogeneous phenotypes (Snow and Judd, 1987). Given the importance of activation kinetics, it may be desirable to express fluorescent reporters that are more rapidly responsive or less persistent in order to support optimized analysis over longer or shorter timescales. Use of reporter proteins with controllable degradation tags may offer potential to this end. While the highest fidelity *in situ* screen would require the use of primary T cells, we expect primary cells to be more functionally heterogeneous than the J76-TPR cell line. While NF-κB and NFAT activation bears functional relevance, the genotype-phenotype coupling of this readout is rather indirect, with many possible factors capable of influencing activation phenotypes other than the CAR sequence. Collectively, these factors point to the likelihood that while *in situ* screens are likely to offer advantages in throughput relative to clone by clone strategies, they are unlikely to achieve the excellent performance attributes used in screens of simpler activities such as protein-protein binding affinity.

Nonetheless, our data demonstrate that NF-κB and NFAT reporter cells are able to identify high-signaling CAR candidates present at low frequencies, and highlight the high potential of *in situ* screening strategies for the development of novel and optimized intracellular CAR domains.

## Data Availability

The raw data supporting the conclusion of this article will be made available by the authors, without undue reservation.
