# Supplementary material for: Toward high-throughput engineering techniques for improving CAR intracellular signaling domains
Source: Front Bioeng Biotechnol. 2023 Mar 27;11:1101122. doi: 10.3389/fbioe.2023.1101122 (PMC10083361; doi:10.3389/fbioe.2023.1101122)
Supplement: Supplementary file 1 [file Table1.DOCX]

Sequences used in CAR constructs


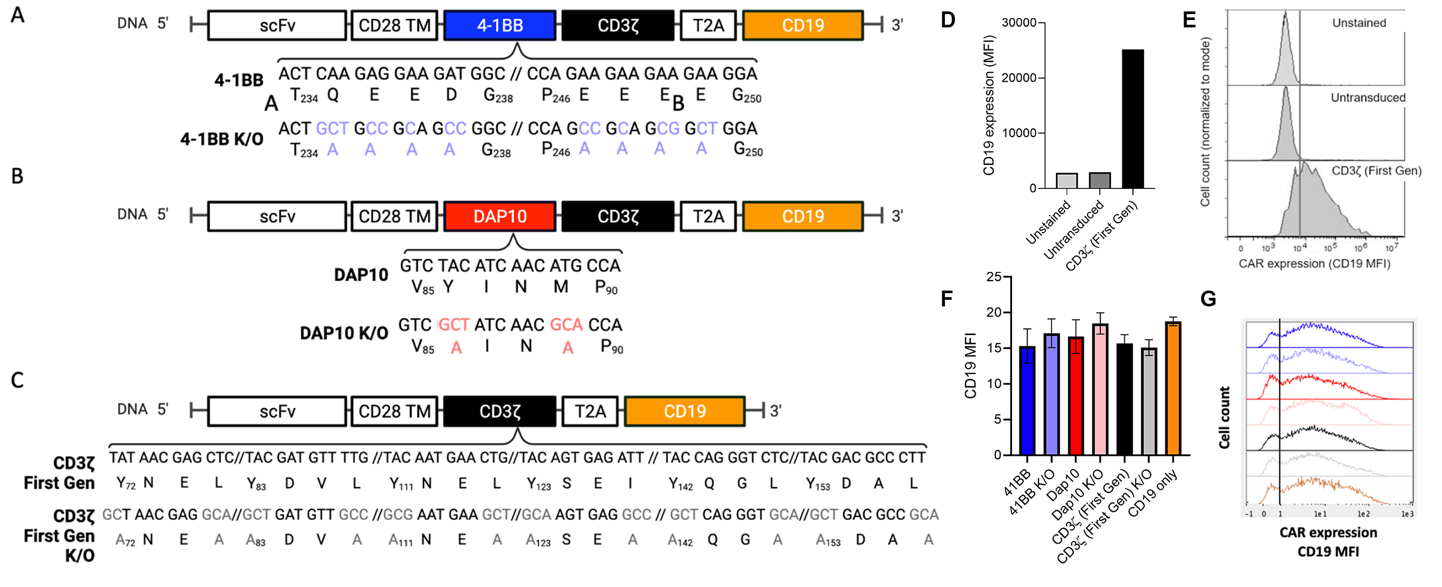


**Anti-human B7H6 scFv derived from TZ47**

CAGGTTCAGCTGCAGCAGTCTGGAGCTGAGCTGATGAAGCCTGGGGCCTCAGTGAAGCTTTCCTGCAAGGCTACTGGCTACACATTCACTGGCTACTGGATAGAGTGGATAAAGCAGAGGCCTGGACATGGCCTTGAGTGGATTGGAGAGATTTTACCTGGAACTGGTAGTACTAACTACAATGAGAAGTTCAAGGGCAAGGCCACATTCACTGCAGATACATCCTCCAACACAGCCTACATGCAACTCAGCAGCCTGACAACTGAGGACTCTGCCATCTATTACTGTGCAATCCCGGGGCCTATGGACTACTGGGGTCAAGGAACCTCAGTCACCGTCTCCTCAGCCGGCGGAGGCGGATCAGGAGGAGGAGGATCAGGCGGAGGAGGATCAGAATTCGACATCAAGATGACCCAGTCTCCATCTTCCATGTATGCATCTCTAGGAGAGAGAGTCACTATCACTTGCAAGGCGAGTCAGGACATTAATAGCTATTTAAGCTGGTTCCAGCAGAAACCAGGGAAATCTCCTAAGACCCTGATCTATCGTGCAAACAGATTGGTAGATGGGGTCCCATCAAGGTTCAGTGGCAGTGGATCTGGGCAAGATTATTCTCTCACCATCAGCAGCCTGGAGTATGAAGATATGGGAATTTATTATTGTCTACAGTATGATGAGTTTCCGTACACGTTCGGAGGGGGGACCAAGCTGGAAATAAAA

**CD28 transmembrane (accession number: NM_001243077.2)**

TTTTGGGTGCTGGTGGTGGTTGGTGGAGTCCTGGCTTGCTATAGCTTGCTAGTAACAGTGGCCTTTATTATTTTCTGGGTG

**4-1BB cytoplasmic (accession number: NM_001561.6)**

AAACGGGGCAGAAAGAAACTCCTGTATATATTCAAACAACCATTTATGAGACCAGTACAAACTACTCAAGAGGAAGATGGCTGTAGCTGCCGATTTCCAGAAGAAGAAGAAGGAGGATGTGAACTG

**DAP10 cytoplasmic (accession number: NM_014266.4)**

AGGAGTAAGAGGAGCCTGTGCGCACGCCCACGCCGCAGCCCCGCCCAAGAAGATGGCAAAGTCTACATCAACATGCCAGGCAGGGGCAAG

**CD3ζ (accession number: NM_001378516.1)**

CTTAGAGTGAAGTTCAGCAGGAGCGCAGACGCCCCCGCGTACCAGCAGGGCCAGAACCAGCTCTATAACGAGCTCAATCTAGGACGAAGAGAGGAGTACGATGTTTTGGACAAGAGACGTGGCCGGGACCCTGAGATGGGGGGAAAGCCGAGAAGGAAGAACCCTCAGGAAGGCCTGTACAATGAACTGCAGAAAGATAAGATGGCGGAGGCCTACAGTGAGATTGGGATGAAAGGCGAGCGCCGGAGGGGCAAGGGGCACGATGGCCTTTACCAGGGTCTCAGTACAGCCACCAAGGACACCTACGACGCCCTTCACATGCAGGCCCTGCCCCCTCGC

**T2A**

AGAGCCAAAAGGTCTGGCTCCGGTGAGGGCAGAGGAAGTCTTATAACATGCGGTGACGTGGAGGAGAATCCCGGCCCT

**CD19 extracellular and transmembrane (accession number: NM_001178098.2)**

ATGCCACCTCCTCGCCTCCTCTTCTTCCTCCTCTTCCTCACCCCCATGGAAGTCAGGCCCGAGGAACCTCTAGTGGTGAAGGTGGAAGAGGGAGATAACGCTGTGCTGCAGTGCCTCAAGGGGACCTCAGATGGCCCCACTCAGCAGCTGACCTGGTCTCGGGAGTCCCCGCTTAAACCCTTCTTAAAACTCAGCCTGGGGCTGCCAGGCCTGGGAATCCACATGAGGCCCCTGGCCATCTGGCTTTTCATCTTCAACGTCTCTCAACAGATGGGGGGCTTCTACCTGTGCCAGCCGGGGCCCCCCTCTGAGAAGGCCTGGCAGCCTGGCTGGACAGTCAATGTGGAGGGCAGCGGGGAGCTGTTCCGGTGGAATGTTTCGGACCTAGGTGGCCTGGGCTGTGGCCTGAAGAACAGGTCCTCAGAGGGCCCCAGCTCCCCTTCCGGGAAGCTCATGAGCCCCAAGCTGTATGTGTGGGCCAAAGACCGCCCTGAGATCTGGGAGGGAGAGCCTCCGTGTCTCCCACCGAGGGACAGCCTGAACCAGAGCCTCAGCCAGGACCTCACCATGGCCCCTGGCTCCACACTCTGGCTGTCCTGTGGGGTACCCCCTGACTCTGTGTCCAGGGGCCCCCTCTCCTGGACCCATGTGCACCCCAAGGGGCCTAAGTCATTGCTGAGCCTAGAGCTGAAGGACGATCGCCCGGCCAGAGATATGTGGGTAATGGAGACGGGTCTGTTGTTGCCCCGGGCCACAGCTCAAGACGCTGGAAAGTATTATTGTCACCGTGGCAACCTGACCATGTCATTCCACCTGGAGATCACTGCTCGGCCAGTACTATGGCACTGGCTGCTGAGGACTGGTGGCTGGAAGGTCTCAGCTGTGACTTTGGCTTATCTGATCTTCTGCCTGTGTTCCCTTGTGGGCATTCTTCATCTTCAAAGAGCCCTGGTCCTGAGGAGGAAAAGAAAGCGAATGACT
